# Supplementary material for: A multi-scale study of thalamic state-dependent responsiveness
Source: PLoS Comput Biol. 2024 Dec 13;20(12):e1012262. doi: 10.1371/journal.pcbi.1012262 (PMC11676947; doi:10.1371/journal.pcbi.1012262)
Supplement: S1 Appendix — Details about employed formulae (Section A), stimuli (Section B), dynamical analysis (Section C), and numerics/software (Section D). Supporting/extending results for the firing adaptation metric (Section E) and for spindle mechanism (Section F). Additional tables and figures. (PDF) [file pcbi.1012262.s001.pdf]

S1 Appendix

A multi-scale study on thalamic state-dependent responsiveness

Jorin Overwiening, Federico Tesler, Domenico Guarino, Alain Destexhe

A Additional formulae

Transfer function

The transfer function F (8) was defined as the complementary error function

$$\mathrm{erf}(z) = 1 - \mathrm{erfc}(z) \equiv \frac{2}{\sqrt{\pi}} \int_0^z \mathrm{d}t \; \mathrm{e}^{-t^2},$$

with $\mathrm{erf}\colon \mathbb{R} \to (-1, 1)$. The statistical interpretation is that for a normal distributed random variable ξ with $\langle \xi \rangle = 0$ and $\langle \xi^2 \rangle = 1/\sqrt{2}$, $\mathrm{erf}(z)$ is the probability of $\xi \in [-z, z]$.

Mean-field correlation

The differential equation describing the correlation of populations $\{\mu, \nu, \lambda\} \in \{e, i\}$ to close the second order statistical moments of the mean-field formalism is given by

$$T \partial_\tau C_{\mu\nu}(\tau) = (\partial_\lambda F_\nu \cdot \nu^0 - \delta_{\lambda\nu}) C_{\mu\lambda}(\tau),$$

where τ is the time lag and ν^0 marks the stationary solution of the firing rate.

B Details on stimuli

Gaussian stimulus

The *split-Gaussian* stimulus employed as a general sensory input was defined as

$$G(t, t_0) = A\big(\theta(t_0 - t)\mathrm{e}^{\frac{-(t-t_0)^2}{2\sigma_l^2}} + \theta(t - t_0)\mathrm{e}^{\frac{-(t-t_0)^2}{2\sigma_r^2}}\big),$$

where $\theta(t)$ is the Heaviside step-function, and with A the amplitude and $\sigma_{l,r}$ the slopes of the left and right side of the split-Gaussian, respectively.

Synaptic noise model

Ornstein-Uhlenbeck type noise can be defined as

$$\frac{\mathrm{d}x}{\mathrm{d}t} = \theta(\mu - x) + \sigma \frac{\mathrm{d}W}{\mathrm{d}t},$$

where μ is the mean reversion level, θ the mean reversion rate, and σ the amplitude of the standard Wiener process $W(t)$, or white noise.

The conductance noise was implemented in the mean-field via adding the mean of OU-type noise (x) in a time window corresponding to T to the static conductances μ_G (see methods). The resulting conductances with including noise are then:

$$\tilde{\mu}_{G(e,i)} = \mu_{G(e,i)} + \overline{x(t)^T_{(e,i)}}.$$

The specific parameters for $x(t)$ used for the case of synaptic noise case in the simulations are $\mu = 0$nS, $\theta = 5\text{ms}^{-1}$, and for the amplitudes $\sigma_i = 200$nS and $\sigma_e = 60$nS.

C Dynamical analysis

The steady and equilibrium points of the spiking network are taken as the averaged population activity over long simulation times for constant inputs where oscillations are ruled out either by observation or by spectrum analysis. For the mean-field these are calculated as the populations activity after a transient up to a point where the Jacobian (J) eigenvalues are negative. The Jacobian is defined on the variable set $X \in \{\nu_e, \nu_i, \omega_e, \omega_i\}$ and calculated via numerical derivatives.

For the bifurcation diagram (Fig. 7d) the same technique was used up to the bifurcation point, except that now $X \in \{\nu_e^1, \nu_e^2, \nu_i^1, \nu_i^2, \omega_e^1, \omega_e^2, \omega_i^1, \omega_i^2\}$. For the mean-field the Jacobian eigenvalues are complex up to the bifurcation point, suggesting a supercritical Andronov-Hopf bifurcation. After the bifurcation point for the spiking network, instead of the averaged firing rates, the extrema are averaged over a long time simulation, mimicking the real sub-plane of the suggested complex-space Andronov-Hopf bifurcation. The same was done for the mean-field.

The maximum Lyapunov exponents are calculated employing the Rosenstein algorithm [1].

D Numerical simulations

All simulations were conducted in `Python`. The spiking network simulations additionally used the `Python` package `Brian2` [2]. Constructing the network, all neuron connections to random to ensure a statistical model comparable to the mean-fields assumptions. All numerical integrations in spiking network simulations employed *Heun's method*, while for mean-field simulations *Euler's method* was used.

E Firing adaptation

We introduce firing adaptation $\mathcal{F}$ as a metric describing how adaptation shapes the response. Because the AdEx generates bursting-like behaviour via its adaptation current ω, we will see a good agreement between $\mathcal{F}$ and burstiness.

We use the transfer function F (8). For the effect of adaptation mechanisms on the firing rate of a neuron type we can identify two crucial states described by F: The no-adaptation fixpoint (F_0): This state represents the firing rate of a cell in the absence of adaptation and corresponds to the cell's firing rate at the onset of a stimulus where the

(slow) adaptation mechanisms have no impact yet. And the real-adaptation fixpoint (F_ω): Representing the cell's firing rate when it has fully adapted to its own and the stimulus influence. Then $F_0 - F_\omega$ reflects the change in firing rate of a cell from initial activity at the onset of a stimulus as it transitions towards full adaptation and slowing its firing rate.

Necessarily, the transfer function is firing-based and needs non-zero firing rate inputs (ν_e, ν_i) to yield results. Therefore, we calculate the fixpoints for a constant $\nu_e = 1$Hz. When considering high firing rates as decreased ISIs this metric is comparable to experimental methods measuring burstiness (such as [3]). Concluding, we define the firing adaptation metric as

$$\mathcal{F} \equiv \frac{F_0 - F_\omega}{F_0}, \quad \text{where} \quad \partial_t F_{\{0,\omega\}}(\nu_e = 1\text{Hz}) = 0.$$

With this, we investigate the stability of bursting in TC cells and the dependence of bursting on model parameters. For this we use the level of firing adaptation to quantify the effect of adaptation mechanisms on the firing rate of neurons. The dependence of TC firing adaptation on membrane and spiking adaptation parameters and membrane polarization is shown in Fig. Aa,b. Firing adaptation is strongest at high membrane adaptation levels and hyperpolarized membrane potentials. The awake state experiences nearly no firing adaptation, while the sleep state shows strong firing adaptation.

We want to improve on defining the states of ACh as tonic/bursting states and subsequently as awake and sleep states. For this we conducted a parameter scan using spiking network simulations of single TC cells mapping the different firing modes. Similar to Fig 1 we injected the cell with a constant current for 1s. We classified firing patterns based on the number of spikes on a time scale relative to the adaptation time constant ($\tau_\omega \simeq 200$ ms). The external current applied was proportional to E_L in order to induce activity (with $I = \{200\text{nA for } E_L = -50\text{mV}, 400\text{nA for } E_L = -85\text{mV}\}$). Fig. Ad presents the results, highlighting the four possible firing modes. The ACh-absent or sleep state exhibits stable bursting not susceptible to either adaptation or voltage perturbations while the ACh-present or awake state is deep in the tonic regime.

The scan shows a similar tendency of increased bursting as with increased firing adaptation. Note that the firing adaptation is not taking into account if there is actually a non-zero response to account for the single spikes or no activity response types of the single cell scan (Fig. Ad). When integrating with $\mathcal{F}$ the actual response amplitude we get however the same strip-region of bursting as in the single-cell scan (see Fig. Ac, the same holds for the scan in b).

F Spindle mechanism

Spindle oscillations are one of the main activity dynamics of the thalamus during slow-wave sleep or anesthesia [4], strongly influencing the responsiveness of the thalamus in such states. These originate from the superposition of multiple cellular and circuit properties. In ACh depleted conditions such as during sleep, T-channel currents promote bursts after hyperpolarization (as modeled in [5] for a Hodgkin-Huxley model),

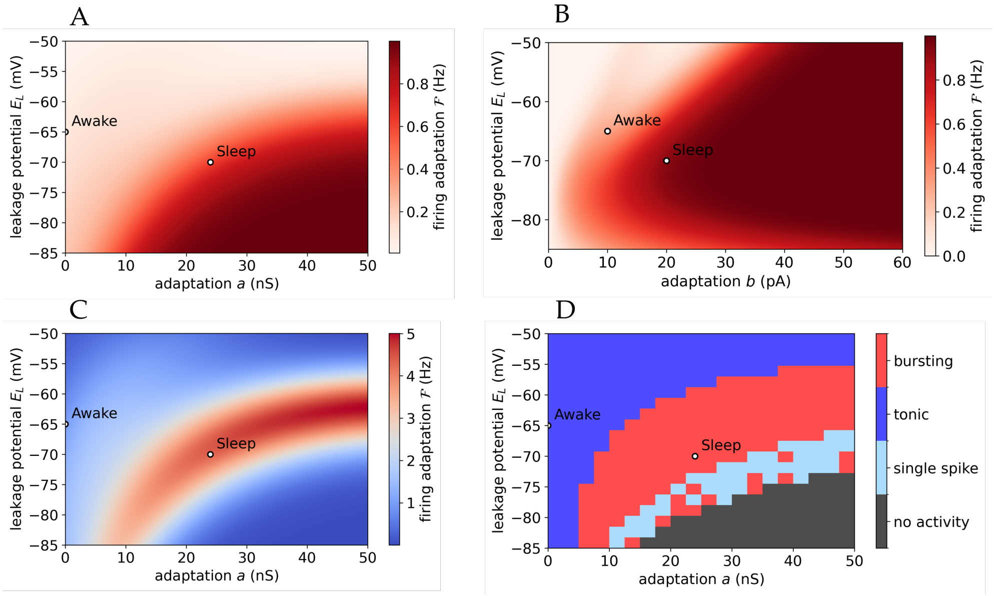

Fig. A Firing adaptation and dynamics of TC cells. A Parameter scan for firing adaptation $\mathcal{F}$ for TC cells via the fitted transfer function (8) for resting potential and membrane adaptation a. Depicted are the parameter values for sleep and awake state. **B** Same as in A but for spiking adaptation b. **C** The scan of A but the firing adaptation $\mathcal{F}$ is multiplied by the firing rate during the response (also via transfer function), revealing a split of bursting dynamics for the same input. **D** Parameter scan of TC single-cell simulations, showing the possible firing modes of tonic, bursting, single spikes, or no spikes as a response to a input current (see text). Similarity to firing adaptation in A and C is evident.

and barrages of IPSPs occur in strongly connected subgroups within the TRN [6]. This creates two self-sustained loops: an inhibitory self-loop within TRN neurons, and a loop connecting RE and TC cells (see colored connections in Fig. Be). The rebound bursts caused by this circuit arrangement generate spindle oscillations, as shown by experiments and computational models [4, 7].

Computational models demonstrated that a 4-neuron structure of TC and RE neurons exhibits self-sustained oscillations with all characteristics of spindles, as shown first for Hodgkin-Huxley type models [8] and later using AdEx models [9]. These oscillations rely on a loop where a TC cell triggers a burst in an RE cell which, in turn, causes a rebound burst in another TC cell, completing the cycle. In their study however the oscillations waned with increased network size.

In this study, we showed that the proposed models in a sleep-like state indeed show spindles at all scales, validating them as thalamic models. In contrast to a previous study [9], we aimed for robust spindles at population scales. To enhance rebound bursting we promoted burst firing by adjusting the reset membrane potential (V_r) below the sodium spike threshold onset: $V_r = -48$mV for TC and $V_r = -42$mV for RE cell. This yields sustained burst firing without sustained activation, mimicking T-channel like activation and IPSP barrages in RE cells. Accordingly we re-calibrated

the mean-field fit to accommodate the change in V_r. (The original fit produces spindles with unrealistic amplitudes.) The resulting fit parameters are depicted in Table B.

The Jacobian eigenvalues calculated at the steady points of the mean-field up to the bifurcation point are complex. This together with the transition into a stable limit cycle suggests a supercritical Andronov-Hopf bifurcation as the origin of spindles (Fig. 7d).

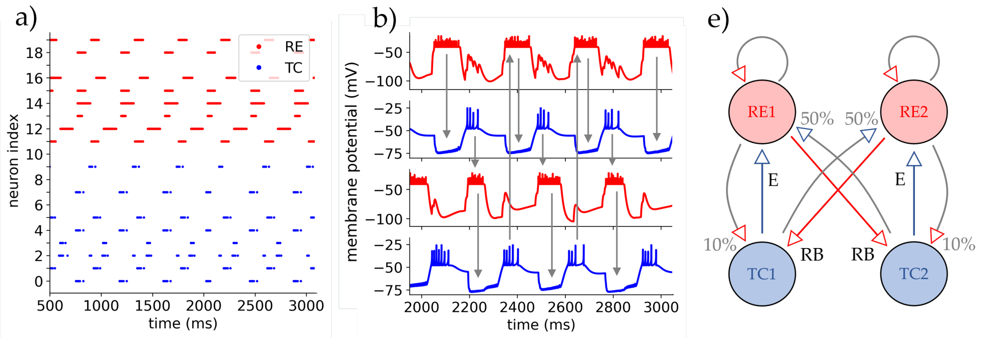

Fig. B Spindle oscillations are generated by sparse TC-RE connections. (a) Raster plot of a simulation for the small network showing all 20 neurons. **(b)** Four neurons showing anti-phase burst firing with rebound bursts of the TC cells as a possible generator of spindles. Cell traces are taken from the simulation in (a) with a small network ($N = 20$). The arrows show the loop of activity propagation between RE and TC cells with rebound bursts and direct excitation. These pathways translate to the four colored connections in (e). **(e)** The 4-structure used for the mean-field connections. The colored arrows mark the main pathway of propagation with rebound bursts (RB) and direct excitation (E) as transmitters. The non-significant connections are marked in grey with their respective relative strength shown in percentages of the original connection probability. The RE populations keep their local self-loop.

In Fig. Ba oscillatory dynamics in a small network of 20 neurons are illustrated, revealing a generating 4-loop structure inside the network (b). We employed the burst-adjusted sleep parameters but with synaptic parameters from [9]. To invoke activity, an initial kick of Poissonian input of high frequency to only a random subset of neurons was applied.

For the mean-field to produce self-sustained oscillations we employ the 4-structure as seen at single cell level (from [9]) and justify its need also at the population level. This is further supported by studies such as [10]. They showed the mechanism behind the generation and specific frequency of oscillations in the thalamus is the delay in the propagation of activity between different TC neurons. Thalamic relay cell clusters have no connection to themselves and so RE neurons serve as intermitter. The slow adaptation mechanisms governing rebound bursts are therefore generating the slow $\sim$10Hz spindle oscillations.

In order to implement this, we construct two RE and two TC populations and keep all connections active, but reduce the connection probability of links which are antagonistic to this propagation via rebound bursts. This can be seen as two close but locally separated subgroups of relay neurons. The resulting structure is depicted

in Fig. Be. (Note: If the connections are set homogeneous then this network is acting exactly like a mean-field with just two populations.) The main links for the propagation are colored and labeled with their mechanism of propagation: RE induced rebound bursts and TC excitation. To initiate the spindles here also a kick of high activity going to only one or two populations was necessary, in agreement with the spiking network. Additionally, T was set to 15ms to have similar phases and shapes with the spiking network.

The spindle-adjusted sleep state differs quite substantially from the sleep state in the rest of this study. The adapted leakage and reset parameters, modelling T-channel like activation and IPSP barrages to invoke stronger rebound bursts, create this significant difference, suggesting two distinct states. One representing a sleep state similar to N2 in which spindles are observed, while the other could represent a less-deep sleep state [11, ch. 44]. This suggests that the concentration of ACh alone is not sufficient for investigating awake and sleep separation, but the mechanisms behind rebound bursts are specifically important to include. Especially how these connect to ACh and other, in this study neglected, neuromodulators like dopamine, norepinephrine, or serotonin. Additionally, we remark that the shown spindles inherit the correct underlying mechanisms, as evident from experiments, but do show a frequency at the lower end of the usually defined spindle frequency, potentially being more close to Delta waves. All this hints at interesting future work in connecting physiological brain states of awake and sleep with multi-scale thalamic models.

Additional Tables and Figures

Table A Formal requirements and restrictions for the employed approximations of the mean-field formalism.

$\tau_\omega > \tau_m$	Mixed activity regime of the AdEx [12]
$T > \tau_{\text{ac}} \approx 1\text{ms}$	No fluctuations (see Fig. 2c), with τ_{ac} the autocorrelation time
$T < \max(\nu_{\text{AI}})^{-1} \approx 15\text{ms}$	with ν_{AI} the maximum firing rate in the thalamus [13]
$\Delta\nu = (NT)^{-1} \simeq 0.1\text{Hz}$	mean-field resolution [14]
$\tau_\omega \gg T$	ω dynamics are independent of fluctuations in ν [15]
$\tau_m > \tau_{(e,i)}$	dynamics of $v(t)$ are stat. independent of synaptic changes
$\mu_G^s/\sigma_G^s \propto \sqrt{\nu_s} <! 1$	approximation for synaptic fluctuations (10)&(11) [16]
$\langle\tau_{\text{eff}}\rangle/\sigma_\tau \propto \sqrt{\sum_s \nu_s} <! 1$	approximation for membrane time constant [16]
Sparse and random network	$G(N,p)$ Erdos-Renyi model with $p \approx 1/N$ [14]
Activity regime	Asynchronous Irregular (AI) in E-I balance [17, 18]

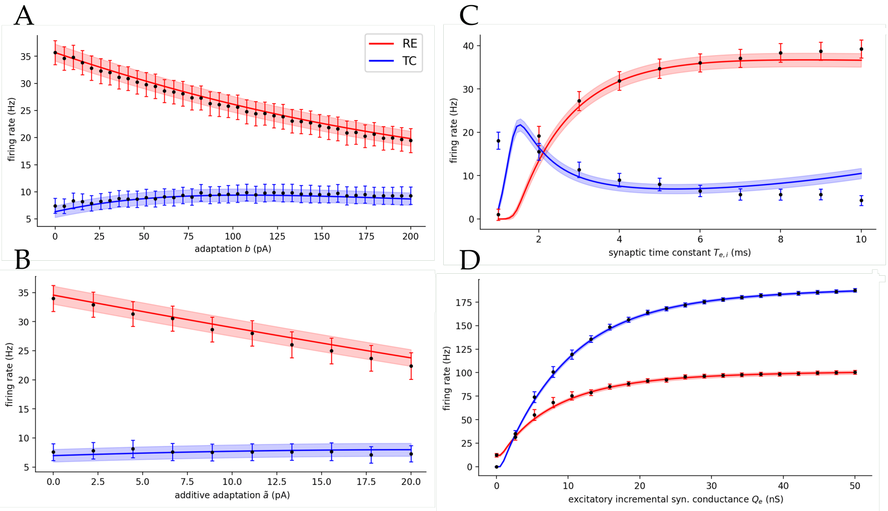

Fig. C Global parameter analysis for mean-field and spiking network. Black markers represent equilibrium population firing rates of the spiking network. Colored line and shaded area represent the mean-fields mean and standard deviation, respectively. **A** Spiking adaptation parameter. **B** Membrane potential adaptation parameter as shift of the original parameters to keep the difference between TC and RE adaptations, securing stable dynamics. A and B ensure the mean-fields fit validity between awake and sleep state. **C** Synaptic exponential delay time constant of both populations. **D** Excitatory incremental synaptic conductance Q_e of the TC population (2). The good fit allows for modeling dynamic clamp-like techniques and suggests that the mean-field is able to capture the non-trivial firing rate saturation of the spiking network (also supported by Fig 2D).

Table B The fitting parameter values for recreating spindles with sleep parameters and included rebound burst mechanism. (All values in mV.)

cell	P_0	P_μ	P_σ	P_τ	$P_{\mu\mu}$	$P_{\mu\sigma}$	$P_{\mu\tau}$	$P_{\sigma\sigma}$	$P_{\sigma\tau}$	$P_{\tau\tau}$
TC	−51.17	3.94	15.53	−7.15	0.35	−7.57	−1.19	−13.61	9.47	29.44
RE	−45.84	3.53	−16.90	41.75	0.34	2.02	−5.23	19.44	49.70	−93.53

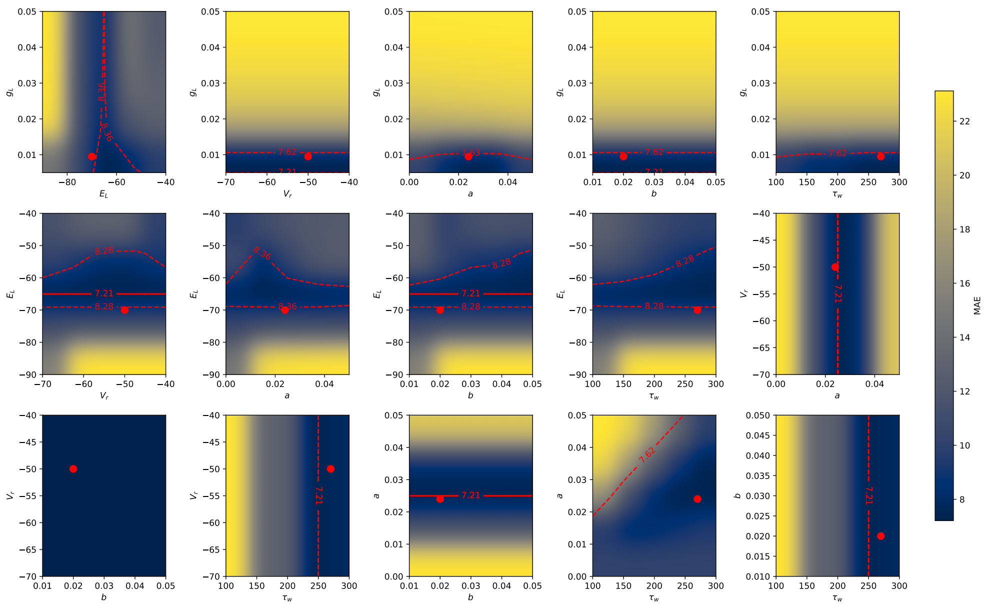

Fig. D Parameter space fit for TC without ACh. The *mean absolute error* (MAE) between the AdEx (1) and the TC cell traces in absence of ACh from [19]. Red lines mark minimum MAE (solid lines) and 5% deviation from said minimum (dashed lines). The employed parameter values are marked as the red dot. (see units in Fig. F)

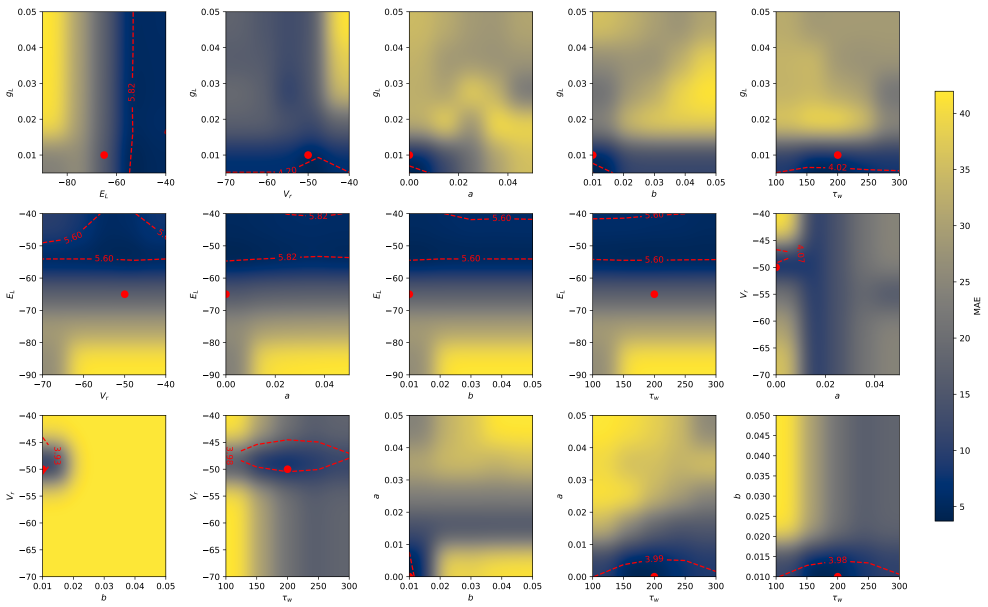

Fig. E Parameter space fit for TC with ACh. (see caption in Fig. D)

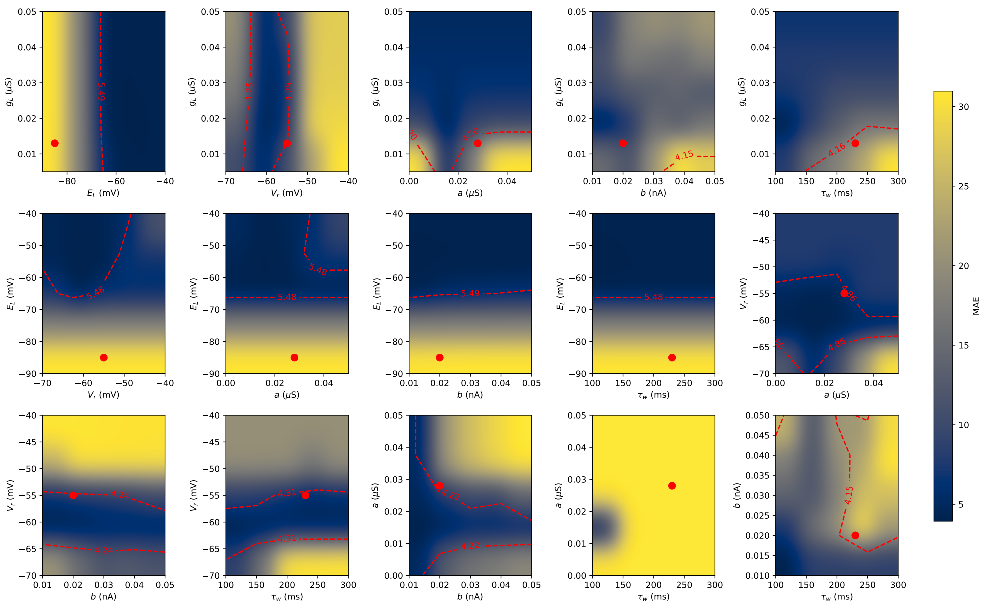

Fig. F Parameter space fit for RE without ACh. (see caption in Fig. D) The chosen value of E_L marks the biggest MAE (middle row). Here we still chose to take a hyperpolarised value to account for the reduced excitability in RE neurons with ACh absent [19] and to inhibit also the population response to keep the stability of the network. (see main text)

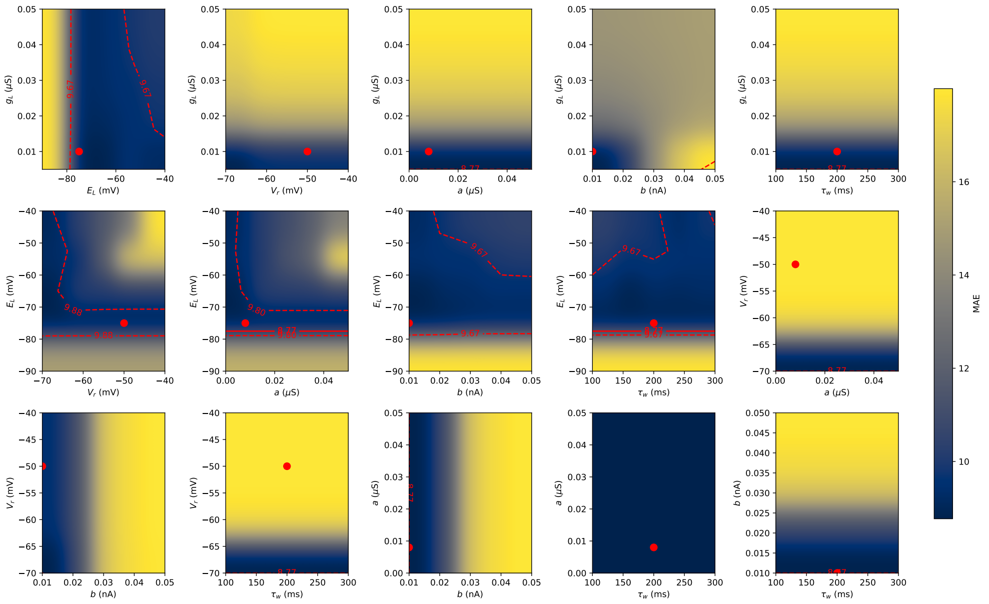

Fig. G Parameter space fit for RE with ACh. (see caption in Fig. D)

References

[1] Rosenstein, M.T., Collins, J.J., De Luca, C.J.: A practical method for calculating largest lyapunov exponents from small data sets. Physica D: Nonlinear Phenomena **65**(1), 117–134 (1993) https://doi.org/10.1016/0167-2789(93)90009-P

[2] Stimberg, M., Brette, R., Goodman, D.F.: Brian 2, an intuitive and efficient neural simulator. eLife **8**, 47314 (2019) https://doi.org/10.7554/eLife.47314

[3] Wolfart, J., Debay, D., Le Masson, G., Destexhe, A., Bal, T.: Synaptic background activity controls spike transfer from thalamus to cortex. Nature Neuroscience **8**, 1760–1767 (2005)

[4] Steriade, M.: Neuronal Substrates of Sleep and Epilepsy. Cambridge University Press, Cambridge UK (2003). https://doi.org/10.1017/CBO9780511541711

[5] Destexhe, A., Contreras, D., Sejnowski, T.: A model of spindle rhythmicity in the isolated thalamic reticular nucleus. J Neurophysiol **72** (1994)

[6] Shu, Y., McCormick, D.A.: Inhibitory interactions between ferret thalamic reticular neurons. Journal of neurophysiology **87 5**, 2571–6 (2002)

[7] Destexhe, A., Sejnowski, T.J.: Thalamocortical Assemblies. Oxford University Press, Oxford UK (2001)

[8] Destexhe, A., Bal, T., McCormick, D.A., Sejnowski, T.J.: Ionic mechanisms underlying synchronized oscillations and propagating waves in a model of ferret thalamic slices. Journal of Neurophysiology **76**, 2049–2070 (1996)

[9] Destexhe, A.: Self-sustained asynchronous irregular states and up–down states in thalamic, cortical and thalamocortical networks of nonlinear integrate-and-fire neurons. Journal of computational neuroscience **27**, 493–506 (2009) https://doi.org/10.1007/s10827-009-0164-4

[10] McCormick, D.A., McGinley, M.J., Salkoff, D.B.: Brain state dependent activity in the cortex and thalamus. Current Opinion in Neurobiology **31**, 133–140 (2015) https://doi.org/10.1016/j.conb.2014.10.003 . SI: Brain rhythms and dynamic coordination

[11] Kandel, E.R., Schwartz, J.H., Jessell, T.M. (eds.): Principles of Neural Science, 3rd edn. Elsevier, New York (1991)

[12] Touboul, J., Brette, R.: Dynamics and bifurcations of the adaptive exponential integrate-and-fire model. Biological cybernetics **99**, 319–34 (2008) https://doi.org/10.1007/s00422-008-0267-4

[13] Sherman, M., Guillery, R.: Exploring the Thalamus and Its Role in Cortical Function. MIT press, Cambridge MA (2009). https://doi.org/10.7551/mitpress/2940.

001.0001

[14] El Boustani, S., Destexhe, A.: A Master Equation Formalism for Macroscopic Modeling of Asynchronous Irregular Activity States. Neural Computation **21**(1), 46–100 (2009) https://doi.org/10.1162/neco.2009.02-08-710 https://direct.mit.edu/neco/article-pdf/21/1/46/818085/neco.2009.02-08-710.pdf

[15] DiVolo, M., Romagnoni, A., Capone, C., Destexhe, A.: Biologically Realistic Mean-Field Models of Conductance-Based Networks of Spiking Neurons with Adaptation. Neural Computation **31**(4), 653–680 (2019) https://doi.org/10.1162/neco_a_01173

[16] Kuhn, A., Aertsen, A., Rotter, S.: Neuronal integration of synaptic input in the fluctuation-driven regime. Journal of Neuroscience **24**(10), 2345–2356 (2004) https://doi.org/10.1523/JNEUROSCI.3349-03.2004 https://www.jneurosci.org/content/24/10/2345.full.pdf

[17] Zerlaut, Y., Chemla, S., Chavane, F., Destexhe, A.: Modeling mesoscopic cortical dynamics using a mean-field model of conductance-based networks of adaptive exponential integrate-and-fire neurons. J Comput Neurosci **45-61**(44) (2018) https://doi.org/10.1007/s10827-017-0668-2

[18] Vreeswijk, C., Sompolinsky, H.: Chaos in neuronal networks with balanced excitatory and inhibitory activity. Science **274**(5293), 1724–1726 (1996) https://doi.org/10.1126/science.274.5293.1724 https://www.science.org/doi/pdf/10.1126/science.274.5293.1724

[19] McCormick, D., Prince, D.: Acetylcholine induces burst firing in thalamic reticular neurones by activating a potassium conductance. Nature **319**, 402–405 (1986)
