# Supplementary material for: A multi-scale study of thalamic state-dependent responsiveness
Source: PLoS Comput Biol. 2024 Dec 13;20(12):e1012262. doi: 10.1371/journal.pcbi.1012262 (PMC11676947; doi:10.1371/journal.pcbi.1012262)
Supplement: Attachment — Submitted filename: ThalamusMeanfield_revision_letter.docx [file pcbi.1012262.s002.docx]

**Revision letter for “A Multi-Scale Study of Thalamic State-Dependent Responsiveness”.**

We thank the reviewers for the helpful comments and suggestions. We provide below a point-by-point answer to their comments.

[Original reviewer’s comments in black and our response below each comment in blue. Changes and revisions in the highlighted manuscript version are marked in red.]

Reviewer #1: In “A Multi-Scale Study of Thalamic State-Dependent Responsiveness” by Overwiening, Tesler, Guarino & Destexhe, the authors present a biological plausible spiking model of the thalamus and a corresponding mean-field method to capture the behavior of the model. The network consists of inhibitory (RE) and excitatory (TC) cells reciprocally coupled with RE self-inhibiting. This modeling study is able to show a variety of known dynamics in these neural networks, including: sleep & awake states with diff params (Table 1) mimicking presence/absence of ACh, linear/nonlinear response of RE depending on source (stim or crtx) and sleep/awake, and effects of synaptic noise to make sleep/wake response less distinguishable. The work is interesting for capturing a lot of thalamic neural activity. They use a mean-field method that requires fitting the transfer function F to simulations of the underlying model in this case (one could use data), but appears to capture many of the details of the simulated model as shown in figures throughout the paper, but appears to be crucial for describing the supercritical Andronov-Hopf bif in the spindle oscillation section (Fig 7d) — is this true?

Major:

1) I am not fully convinced that this mean-field treatment of these models is ‘novel’ (lines 29, 109). Perhaps the application is novel or the fitting procedure to get the effective transfer function is novel but there are an abundance of mean-field type models for recurrent E&I networks, especially when the units are all intrinsically homogeneous, even when there is (slow) adaptation. An easy fix is just getting rid of the ‘novel’ phrase in these 2 places; if the authors insist on keeping ‘novel’ then a long discussion needs to be added for how different this is compared to [22, 47, 50] and the many other related papers.

We agree that the proposed model is not “novel” in the sense of a new mean-field framework. Therefore, we have removed the word novel from line 109. However, the present model is novel in the sense that there is no such model that was proposed previously for the thalamus, under this mean-field framework. So, we have kept the term novel only in the abstract where “novel” refers to this new thalamic implementation: “The development of this novel thalamic mean-field model provides a new tool for incorporating detailed thalamic dynamics in large scale brain simulations.” (lines 29-31).

2) Related to the prior point. [46] is referenced a lot in the Methods but I can’t seem to find it. Please make it publicly available with a link to arXiv or bioRxiv. There is a worry that this paper is only tweaking 2 parameters compared to [46]: the E_L and the b (spike adapt param). Is that the main methodological difference? I am not discounting the biological results that are captured with different parameters as stated in the paper, but the differences between [46] and this paper need to be clearer in this paper, and if appropriate in [46]. In [46] based on the title, the authors are analyzing different neural network responses with and without ACh, which is the main thrust of this paper.

Unfortunately the reference [46] is still in preparation (unpublished). Because of this and to make the current paper more self-contained, we removed the references to [46] and included an explanation of the fit in the text (lines 179-185). To answer the novel/similarity point: We first point out that in [46] the analysis is restricted to single cell dynamics, while our work is focused on the analysis of neuronal networks and the introduction of a mean-field model that describes the neuronal activity at the mesoscale level. In addition, one of the main topics of our paper is the analysis of the response of these neural network to external stimuli under different states (no such analysis will be presented in [46] as it deals only with single-cell dynamics). The only common point between the two works is limited to the initial determination of the single-cell model parameters from experimental data. In addition, in Ref. [46] the analysis is expanded to single-cells from other brain regions beyond the thalamus (notably the basal-ganglia and its multiple nuclei), for which it constitutes a more general analysis on neuromodulators at the single-cell level. Any similarity or overlap between our paper and [46] will be properly mentioned in the final version of reference [46].

3) For the mean-field fitting procedure, it all seems a bit strange i.e., the quadratic form of V_theta^eff in eqn (9). Was the exact same procedure used in [50], or were there differences in the model? Presumably this is just a least-squares fit to the transfer function F that is numerically calculated in the network simulations (mapping grid of nu_e,nu_i inputs to outputs).

We follow the same fitting procedure as in Ref. [48] which combines the works of Refs. [49,50] (line 223-224). In Ref. [50] a transfer function (Eq. 8) with a general phenomenological membrane potential threshold function V_theta^eff (Eq. 9) was proposed. Where V_theta^eff takes the general form of a second order polynomial expansion because there is no theoretical form. The (Eq. 9) depends on the membrane subthreshold statistical moments {mu_v, sigma_v} and in [50] these were extracted from data directly. Ref. [48] proposed to combine this with the analytical form of these moments as functions of input firing rates {nu_e, nu_i} which were derived in [49]. With this the transfer function (8) can be written as a function of {nu_e, nu_i} and be fitted on firing rate data. However, the fit parameters P of (9) have to be found. The fitting procedure itself is then to reorder (8) and get V_theta^eff as a function of {F, nu_e, nu_i}, where F is the output firing rate. On this the polynomial (9) is fitted in first order to find the correct minima. The main fit is then with the full second order polynomial with transfer function on the mapping of input to output firing rates {nu_e, nu_i} -> F (Fig 2a,b), where the previous linear fitting parameters are used as initialization.

4) Figure 3a), bottom panel. Where is the red curve, mean-field of RE? For both Fig 3a) b) bottom panel (populations), why isn’t the simulated network of RE shown? The simulated network of TC is shown in blue.

We wanted to keep the same y-scale for both Fig 3a and b (bottom) for comparison. Because the RE activity is high at ∼ 50Hz and is following exactly the shape of the TC curve with same frequency and phase, we opted to leave it out. Because in the last section we validated the mean-field with the spiking network already we wanted to improve clarity in this figure by only showing the (discussed) TC activity. Nevertheless, we agree that just a missing RE activity without explanation is questionable and we therefore added a brief explanation in the caption of Fig 3: “RE activity is of same frequency and phase as TC activity but with amplitude ∼ 50Hz (not shown)”.

5) Fig 6b is never discussed or referenced in the main text? Is this around lines 550-553? Also, please explain the statement “Remarkably, spike probability is significantly lower than without noise” on line 553, because if this is comparing Fig 6a and 6b, this statement appears true with reasonable ISI (<450ms. is that right?).

We have added a reference to Fig 6b in line 553. The statement compares 6a and 6b but it was unclear. We rewrote the sentence to: “Remarkably, with noise spike probability is significantly lower even with spike summation (ISI->0ms), independent of polarization.”

6) line 127-129. suggest changing ‘numerously’ to ‘has often’. Can also add the reference “how good are neuron models?” 2009 Science by Gerstner & Naud which was actually fit to LGN data driven by visual stim.

We changed ‘numerously’ to ‘has often’ and included the reference. We thank the reviewer for the suggestion.

Minor:

line 39 in abstract, what does “drastically changing its responsiveness” refer to? presumably TC cell firing and/or its transfer function, please clarify.

It refers to the qualitative change in thalamic behavior and especially in TC firing activity (as the main thalamic output) in response to stimuli when going from the non-spindle state to the spindle state. For example, there is self-sustained activity (oscillations) in the spindle state interacting with stimuli. We have modified this statement in the abstract for clarity.

Line 91: not sure what `far of the fitting point’ refers to, I can guess it means that the phenom. models cannot be used to gain insights when params are varied. Please clarify.

Yes, the phenom. models mostly are linear and effectively fitted only at fix points
so that these only work at or close to the fitting point (no deviations from the fitted parameters). Whereas the proposed model proves to work even with substantial parameter changes, allowing to only use one model for different system/parameter states, as is here the case for awake/sleep cases.

Equation (6), cov of 2 pops, but of what? Voltage, W, or firing? Please clarify on line 214. Figured it out after seeing Fig 2e upper right panel & supplementary section S.1 ‘Mean-field correlation’ but it was annoying to hunt it down.

Clarified in the text that the covariance is between two population’s firing activity.

Line 284: ‘of’ -> ‘from’

We have made the modification.

Figure 2 e), if the non-zero width of the distributions in firing & voltage stem from different (random) number of input connections, please state this. My reading of the paper is that the intrinsic params are homogeneous.

The non-zero width distributions of the spiking network are originated by the Poissonian input and, as the reviewer indicates, by the random connectivity of the network. The random connectivity of the network is stated in line 155 from the paper. This distribution in the firing rate and voltage is correctly captured by the second order mean-field model.

When using multi-scale throughout the paper. What is multi-scale? Seems like it is single-cell spiking model to larger (homogeneous) population, but in my opinion this is not really multi-scale, this is just usual mean-field.

In our paper we analyse single-cell activity and parameters (micro-scale), network activity and parameters (meso-scale) and we develop a mean-field model of the population activity. Furthermore, we consider a variety of single-cell recordings under different states and stimulations, and we compare these experiments with single-cell, network and mean-field simulations. Therefore, not only the mean-field is investigated but the mean-field in a meaningful combination with single cell experiments and simulations, justifying the use of the term multi-scale at least on the micro- to meso-scales level.

Reviewer #2: In this manuscript, Overwiening et al., present a clear addition to the growing body of multiscale models across sleep/wake states, with a focus on thalamocortical and thalamic reticular hyperpolarisation-mediated bursting. They found differences in TC output across varying levels of adaptation, sources of drive, and noise. Along with a demonstration of spindle-like activity, via TC/RE interactions. The study is informative and will be of interest to computational modellers and neuroscientists interested in sleep dynamics. Below I outline some very minor comments.

- I suggest incorporating supp S.5 into the main text.

We thank the referee for the suggestion. S.5 was included in the main text in earlier drafts, but we found it more appropriate to move it to the supplementary because 1) the therein described metric is only used for one minor plot (Fig 5d) outside the parameter scans (Fig S1) which are more focused on further validating the transfer function/mean-field than to provide qualitative new results on the main focus (of responsiveness across scales). 2) The content of the main text is quite long already. Thus, to improve the readability of paper we have kept this section in the supplementary materials.

- Clarify how the bifurcation observed in spindles, identified as a Hopf?

A clarification is included in the supplementary material. In the supplementary S3 we provide the following description (lines 918-920): “The Jacobian eigenvalues are complex up to the bifurcation point, suggesting a supercritical Andronov-Hopf bifurcation” and explain above briefly the procedure. Additionally, in the caption of Fig 7 we also state “*suggested* Andronov-Hopf bifurcation” to emphasize this.

- What is the timestep of cellular simulations?

Timestep is 0.1ms for all simulations (single cell, spiking network, and mean-field).

- “The single cell traces in (a) and (b) reproduce the experiments of Sherman and Guillery [7, ch. 6]. “ could this validation be demonstrated/with reproduced figures in fig ½

This validation cannot be demonstrated with Fig 1b because we need the full network (to reproduce the “in vivo” condition) to model the experiment which used a visual stimulus which activated not only the recorded cell but the whole LGN plus TRN. The network simulations in Fig 2 are the same as in Fig 3 but only in awake state, so Fig 3a and Fig 3b use the same network as in e.g. Fig 2c with just different stimuli.

- Line 597: “create steady state AI dynamics, with how- ever spindle oscillations starting as soon as the input stops.” – unclear

We have now changed the wording from “Only prolonged and constant inputs of a duration longer than multiple spindle periods destroy the synchronisation and create steady state AI dynamics, with however spindle oscillations starting as soon as the input stops.” to “Only prolonged and constant inputs of a duration longer than multiple spindle periods destroy the synchronisation and create steady state AI dynamics. However, spindle oscillations emerge again as soon as the input stops.”

- Line 629: “we state as an important validation that the mean-field model is successfully capturing the important nonlinear thalamic feature of bursting. “ – unclear

By this we mean that 1) Bursting is a particularly important feature of the thalamus which according to experiments is crucial for thalamic function and especially for attention/sleep. So it is necessary to have this dynamic in “thalamic” models. 2) Bursting is a highly nonlinear behavior generated by single-cell scale dynamics and defined via spike times. It is not obvious that a rate-based mean-field can capture these effects. To our knowledge there is no study investigating bursting with a mean-field model. We have modified this sentence in the text for clarity.

- Fig. 3 caption “Fig. 3d, there is depicted the peak response amplitude of the thalamus as a function of” Fig. 3e?

We thank the referee for the observation. We have changed Fig 3d to Fig 3e in line 412.

- Reference to fig 6.b is missing in text. A related overall comment, using linking descriptors (e.g. Fig. 6c red/ 6c green) more often would improve the readability of the ms.

We have dded a reference to Fig 6b in line 553.

- Fig 7 caption, repeated (d) entries.

We thank the referee for the observation. We changed the first entry (d) -> (e).

**Have the authors made all data and (if applicable) computational code underlying the findings in their manuscript fully available?**

Reviewer #1: **No:** I would like the authors to make their code publicly available, in particular the fitting procedure for the transfer function F could be a major detail

Reviewer #2: Yes

Next to the already available code for simulating mean-field and spiking network, we also made the code for the described fitting procedure for the transfer function F of the mean-field formalism available in the same GitHub repository (see lines 673-677).

Jorin Overwiening, Federico Tesler, Domenico Guarino, and Alain Destexhe.
