# Supplementary material for: A multi-scale study of thalamic state-dependent responsiveness
Source: PLoS Comput Biol. 2024 Dec 13;20(12):e1012262. doi: 10.1371/journal.pcbi.1012262 (PMC11676947; doi:10.1371/journal.pcbi.1012262)
Supplement: Attachment — Submitted filename: ThalamusMF Revision letter 2_ FT.docx [file pcbi.1012262.s003.docx]

**Revision letter 2 for “A Multi-Scale Study of Thalamic State-Dependent Responsiveness”.**

We thank the reviewers for the comments and suggestions. We provide below a point-by-point answer to their comments.

Original reviewer’s comment in black and our response below in blue.

My final concern remains with the use of the phrase novel, it is simply not needed and should be removed from the text/abstract.

The work is a nice exploration without needing to claim novelty, and it only detracts from the work ignoring previous mean-field implementations of thalamocortical dynamics (unless you add a detailed paragraph unpacking the precise novelty). For example, here is a mean field thalamocortical model from a decade ago with thalamic bursting (https://doi.org/10.1016/j.jtbi.2015.01.028) that should be referenced.

To avoid any confusion, we have now removed the word ‘novelty’ from the abstract. We have also added a citation to the paper mentioned by the reviewer and to a related work presented in Robinson et al, 2008 (<https://www.sciencedirect.com/science/article/abs/pii/S0022519307005164>). However, we notice that in these previous works they only consider bursting from thalamic reticular neurons, but not bursting of relay neurons as in our model. Additionally, their work is focused on the generation of specific types of dynamics (such as slow waves) without going into details about state transitions, thalamic responsiveness, or the analysis of the role of neuromodulators. Whereas in our model these elements are taken into account and studied in detail.

We have now included the following paragraph in the Discussion section addressing the differences between our paper and these previous works:

“Existing mean-field models of thalamocortical dynamics [22, 23], have previously incorporated thalamic single-cell dynamics, such as bursting, into mesoscopic models. While these previous models provide a valuable approach for studying thalamic states, they have included so far only bursting from reticular cells, while bursting from relay neurons was not taken into consideration. In addition, these previous works were focused on reproducing specific types of dynamics (such as slow-waves), while more extensive analysis regarding the transition between thalamic states and the response to sensory stimulation was not investigated. Finally, the key role of neuromodulators in the transition between thalamic neuronal dynamics and states was not captured by these models, which is central to understand the mechanisms behind these transitions. In our work we presented a detailed study reproducing multiple neuronal dynamics found in the thalamus (such as spindles, asynchronous irregular activity and different response modes). Furthermore, these different dynamics emerge as a result of the action of neuromodulators at the single-cell level that are captured within our mean-field model by following a bottom-up approach.”

Jorin Overwiening, Federico Tesler, Domenico Guarino, and Alain Destexhe.
